# The suppression of a selfish genetic element increases a male's mating success in a fly

**DOI:** 10.1002/ece3.10719

**Published:** 2023-11-12

**Authors:** Sophie Lyth, Andrea J. Betancourt, Tom A. R. Price, Rudi L. Verspoor

**Affiliations:** ^1^ Institute of Infection Veterinary and Ecological Sciences, University of Liverpool Liverpool UK; ^2^ Institute of Systems Molecular, and Integrative Biology, University of Liverpool Liverpool UK

**Keywords:** female preference, mate choice, mating behavior, meiotic drive, selfish genetic elements, suppression

## Abstract

X chromosome meiotic drive (XCMD) kills Y‐bearing sperm during spermatogenesis, leading to the biased transmission of the selfish X chromosome. Despite this strong transmission, some natural XCMD systems remain at low and stable frequencies, rather than rapidly spreading through populations. The reason may be that male carriers can have reduced fitness, as they lose half of their sperm, only produce daughters, and may carry deleterious alleles associated with XCMD. Thus, females may benefit from avoiding mating with male carriers, yielding a further reduction in fitness. Genetic suppressors of XCMD, which block the killing of Y sperm and restore fair Mendelian inheritance, are also common and could prevent the spread of XCMD. However, whether suppressed males are as fit as a wild‐type male remains an open question, as the effect that genetic suppressors may have on a male's mating success is rarely considered. Here, we investigate the mating ability of XCMD males and suppressed XCMD males in comparison to wild‐type males in the fruit fly *Drosophila subobscura*, where drive remains at a stable frequency of 20% in wild populations where it occurs. We use both competitive and non‐competitive mating trials to evaluate male mating success in this system. We found no evidence that unsuppressed XCMD males were discriminated against. Remarkably, however, their suppressed XCMD counterparts had a higher male mating success compared to wild‐type controls. Unsuppressed XCMD males suffered 12% lower offspring production in comparison to wild‐type males. This cost appears too weak to counter the transmission advantage of XCMD, and thus the factors preventing the spread of XCMD remain unclear.

## Graphical abstract

We investigated the mating ability of SR, suppressed SR, and wild‐type males. We found that males that carried a suppressor against SR had higher mating success. We also found that SR suffered a fitness cost in terms of reduced offspring production.

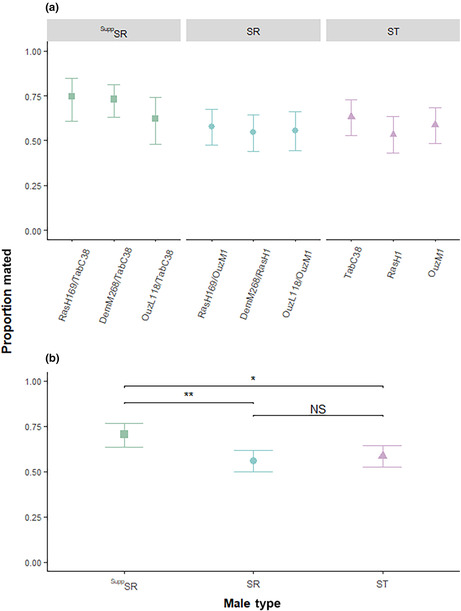

## 1 INTRODUCTION

Choosing the wrong mate can have profound effects on a female's fitness (Trivers, 1972). Females often have high investment in choosing a high‐quality mate that can either directly or indirectly increase her reproductive success (Oneal et al., 2007). For example, mating with the right male can give access to a higher quality territory (Hasegawa et al., 2012), increased fecundity through nuptial gifts (Wedell & Ritchie, 2004), increase the number of offspring she can produce (Andersson & Simmons, 2006) or by ensuring her offspring have high genetic quality (Byers & Waits, 2006; Suzaki et al., 2013). In many species, males can have certain traits that make them low in quality. These include age (Avent et al., 2008; Verspoor, Cuss, & Price, 2015), carrying sexually transmitted diseases (Hurst et al., 1995), having low fitness genes (Lesna & Sabelis, 1999), losing offspring to genetic incompatibilities (Verspoor et al., 2018), or carrying selfish genetic elements (SGEs; Burt & Trivers, 2009). Mating with males that carry any of these traits can be costly for females, and so there should be selection for females to avoid them if they can distinguish between high‐ and low‐quality mates (Jennions & Petrie, 1997).

One widespread category of element that can result in the low fitness of males is SGEs (Verspoor et al., 2020). SGEs subvert the laws of fair Mendelian inheritance and can bias their transmission into subsequent generations, often at a cost to the rest of the genome (Burt & Trivers, 2009). Thus, SGEs can spread without increasing the fitness of the organism that carries them. SGEs are near ubiquitous in nature (Hurst & Werren, 2001) and include transposable elements, homing endonucleases, and meiotic drivers; endosymbionts, although they can be beneficial to their hosts, can also share characteristics of SGEs. SGEs are often costly to the individuals who carry them and so there is a benefit for females to evolve mating behavior that will disfavor SGE‐bearing males (Lande & Wilkinson, 1999; Lindholm et al., 2016; Reinhold et al., 1999; Tregenza & Wedell, 2000). Genetic suppressors are a common evolutionary response to SGEs and endosymbionts (Bastide et al., 2011; Hornett et al., 2006, 2008; Tao et al., 2007; Verspoor et al., 2018, 2020). Suppressors have the ability to block the mechanisms that allow SGEs to gain a transmission advantage, and consequently fair Mendelian inheritance is restored. There can be ongoing co‐evolution between SGEs and their suppressors that could impact the ability and the need for females to discriminate against SGE‐carrying males. However, the question of whether a male carrying a suppressed SGE is as fit as a wild‐type male still remains.

Females have evolved responses or preferences to prevent them from mating with SGE carriers in some cases. In stalk‐eyed flies, females preferentially mate with males with wider eye spans, whereas SGE‐carrying males are generally associated with smaller eye spans (Cotton et al., 2014; Wilkinson et al., 1988). The presence of sperm‐killing meiotic drive also correlates with the rate of polyandry in populations, where meiotic drive is more common in areas with low polyandry (Pinzone & Dyer, 2013; Price et al., 2014). High rates of polyandry can reduce the chance that an SGE‐carrying male will father a female's offspring due to them being poorer sperm competitors (Policansky & Ellison, 1970; Price, Bretman, et al., 2008). Indeed, higher rates of polyandry can evolve in lab populations when they are exposed to an SGE (Price, Hodgson, et al., 2008). Finally, the presence of SGEs and endosymbionts can promote female avoidance behaviors. Carriers of symbionts that can induce cytoplasmic incompatibilities can be prevented by female mate avoidance (Miller et al., 2010). Additionally, female house mice show mate avoidance from males that carry the incompatible *t* haplotype based on olfactory cues; however, this behavior is not always replicated (Manser et al., 2015; Sutter & Lindholm, 2016). Despite this, reports of female discrimination against SGEs remain surprisingly rare (Price & Wedell, 2008), highlighting that female mating responses or discrimination are not uniform or not always found when looked for.

Perhaps an under‐examined factor in the search for female choice against SGEs is the interplay between SGEs and their suppressors. Suppressors may be linked to their own signals that promote female choice or null any effects created by SGEs. Yet, the influence of SGE suppressors on mating behavior has not been widely examined. This is surprising as SGEs and their suppressors can affect gonad development (Bradshaw et al., 2022; Lyth et al., 2023 in review; Meade et al., 2020), which could impact male quality and fertility.

One well‐known SGE that has profound effects on male carriers is X chromosome meiotic drive (XCMD). *Drosophila subobscura* harbors an XCMD system (Jungen, 1967), henceforth referred to as “SR”, which we use as the model for this study. XCMD manipulates the process of meiosis in males where selfish X chromosomes kill Y‐bearing sperm during spermatogenesis, resulting in the production of only daughters (Jaenike, 2001). The transmission advantage gained by XCMD can allow it to rapidly spread through populations, distorting sex ratios, and can even lead to population extinction owing to a lack of males (Hamilton, 1967; Price et al., 2010). However, SR appears to have remained stable at approximately 20% in North Africa for at least 50 years (Hauschteck‐Jungen, 1990; Jungen, 1967; Verspoor et al., 2018). The lack of its spread suggests that SR carries costs strong enough to counterbalance its transmission advantage.

Mating with XCMD males can impose significant costs to females in multiple ways. (1) Males that carry XCMD lose half of their ejaculates, so males may transfer fewer sperm (Angelard et al., 2008; Policansky & Ellison, 1970; Price, Bretman, et al., 2008). This may in turn reduce the number of offspring a female can produce. (2) XCMD tends to occur in areas of low recombination, such as inversions (Jaenike, 2001). The reduced recombination can allow the accumulation of deleterious mutations that can reduce a carriers fitness (Finnegan et al., 2019; Keais et al., 2020; Larner et al., 2019; Wilkinson et al., 2006). (3) A female that mates with an XCMD male produces a female‐biased brood (Hamilton, 1967). This becomes costly in a female‐biased population where the value of producing females is reduced. These costs of mating with an XCMD carrier suggest that it will be beneficial for a female to avoid mating with carrier males. Additionally, there is also suppression of SR (Verspoor et al., 2018), which makes SR in *D. subobscura* a good model to investigate if suppressors themselves alter mating behavior. Unexpectedly, it appears that the suppressor has not spread through populations as it remains rare at a frequency of 1–5% in populations across North Africa (Verspoor, R.L., Lyth, S., & Price, T.A.R.,  unpublished data), suggesting that the suppressor may also be costly in itself. Interestingly, suppression of XCMD in the SR system is also associated with abnormal testes size and even visible abnormalities to testes (Lyth et al., 2023 in review), which could in itself influence the fitness of those males that carry both XCMD and suppressors.

In this study, we look for effects of carrying SR and/or its suppressor on mating behavior. We compare the mating ability of SR males (that carry a driving X chromosome) and males that carry suppressed SR (^Supp^SR) in comparison to standard (ST) males that do not carry a driving X chromosome. This will first allow us to determine whether SR males are inferior to ST males or whether SR bears no detectable mating costs. It will also allow us to investigate whether ^Supp^SR has its own associated fitness costs or whether the fitness of these males is comparable to ST. The costs of suppressors are rarely considered in XCMD studies. Finally, we look at males that carry the suppressor genes in the absence of SR to see if they suffer fitness costs in comparison to males that carry susceptible genes in the absence of SR. Any costs found of SR or its suppressor may provide insight into why SR or the suppressor have not become fixed in populations.

## 2 MATERIALS AND METHODS

### 2.1 Origin and maintenance of fly stocks

We used isofemale lines (Parsons & Hosgood, 1967) of *Drosophila subobscura* established from flies collected in Tabarka, Tunisia (abbreviated to Tab) (36.95° N 8.74° E) in 2013 (Verspoor, Cuss, & Price, 2015), Ras el Oued, Morocco (abbreviated to Ras) (34.15° N 4.00° W), Demnat, Morocco (abbreviated to Dem) (31.73° N 7.01° W), and Ouzoud, Morocco (abbreviated to Ouz) (32.02° N 6.72° W) (Lyth et al., 2023 in review), using traps baited with banana, beer, and yeast. Flies were collected from these locations as it is representative of the range of *D. subobscura* populations across North Africa and locations were separated by a distance of 1400 km. Wild‐caught females were used to establish standard isofemale lines (henceforth referred to as “isolines”), derived from her inbred descendants. All isolines were kept in standard *Drosophila* vials on an ASG double yeast medium (10 g agar, 85 g sucrose, 40 g yeast extract, 60 g maize, 1000 mL H_2_O, 25 mL 10% nipagin) at 18°C on a 12:12 light/dark cycle.

Experimental flies were generated by collecting virgin flies from each isoline on a 24‐h cycle, to ensure no mating had already taken place (Holman et al., 2008). Fly collection was done using CO_2_ anesthesia to separate flies by sex. Flies were stored in new vials on a sugar yeast medium (100 g sugar, 100 g yeast extract, 20 g agar, 1000 mL H_2_O, 25 mL 10% nipagin, 5 mL propionic acid) with no more than 10 flies per vial. All experimental flies were aged to 7 days, as full sexual maturity is reported by this age for both sexes (Holman et al., 2008).

### 2.2 Introgression of SR X chromosomes

**FIGURE 1:**
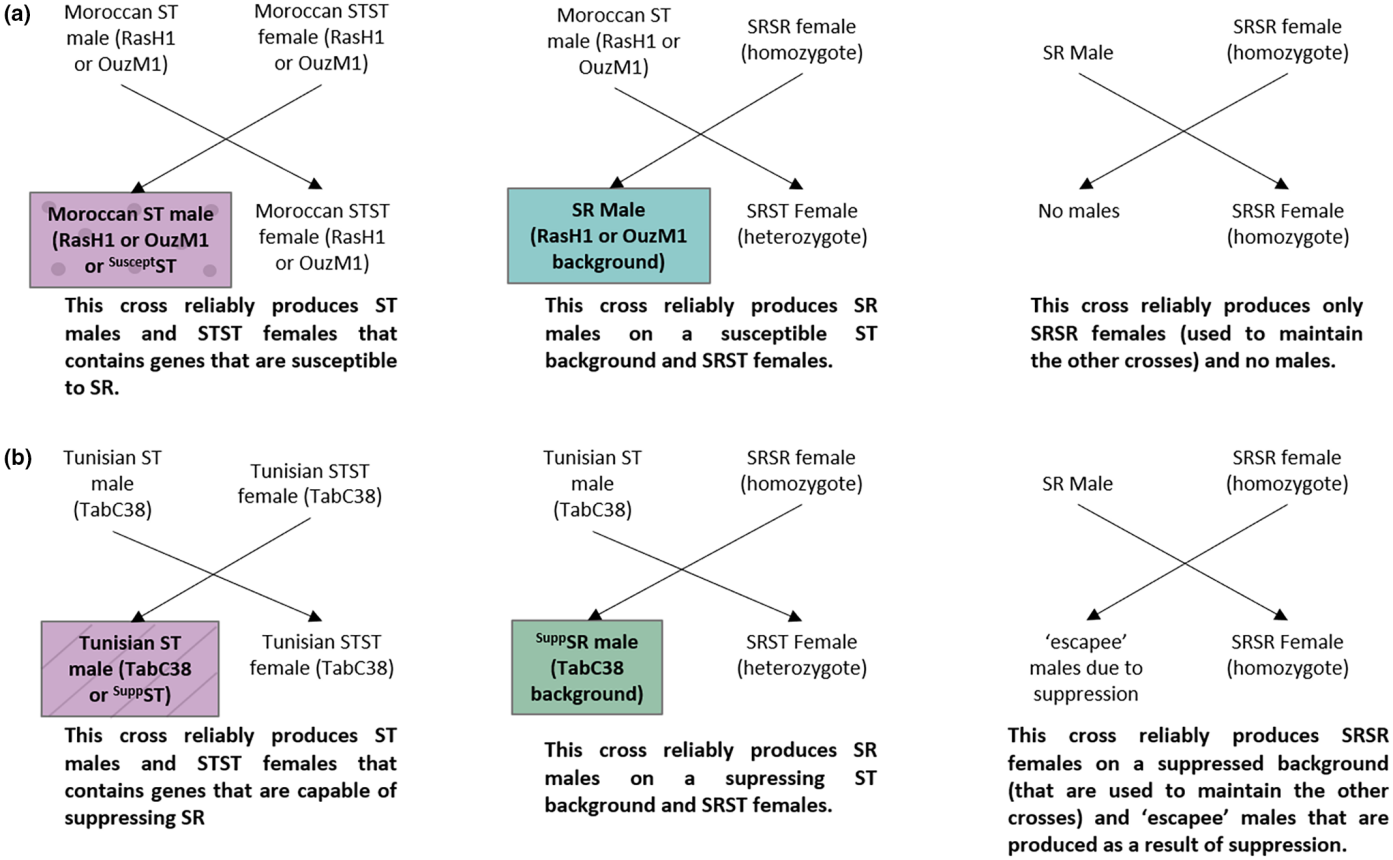
Crossing schematic showing how SR chromosomes were introgressed onto either (a) susceptible ST backgrounds of RasH1 and OuzM1 (sometimes referred to as ^Suscept^SR) or (b) suppressing ST backgrounds of TabC38, sometimes referred to as ^Supp^ST to produce either ST males, SR males, ^Supp^SR males (outlined) used in this study. The male types are also color coded to the subsequent figures as this is how each male type was generated.

For this study, we produced males that differ only in (1) whether or not they carry a driving X chromosome and (2) whether or not the male carries the suppressor genes or ones susceptible to the driving X chromosome. We crossed driving SR chromosomes from three locations in Morocco (referred to as either RasH169, DemM268, or OuzL118) to either suppressing or susceptible genetic backgrounds (the suppressing genetic background will be referred to as TabC38 and the susceptible ones as RasH1 and OuzM1). ST X chromosomes (From TabC38, RasH1, and OuzM1) were also crossed onto identical genetic backgrounds, ensuring that the only difference between males was their X chromosome. Thus, we were able to produce suppressed SR males (^Supp^SR), susceptible SR males (SR), and ST males with a non‐driving X chromosome that drive carriers were compared against (Figure 1).

We introgressed the SR X chromosomes onto ST isoline backgrounds for at least 12 generations. Each generation of introgression required two crosses: First, females homozygous for the SR X chromosome (“SRSR” females) were crossed to an ST male from the target isoline, producing males that carry the SR X chromosome and the target isoline autosomes. Next, these SR males are then crossed to an SRSR females, producing the next generation of introgressed SRSR females (Figure 1). Over time, each of the SR X chromosomes will be introgressed onto the target ST autosomes, where it is expected that by nine generations, 93% of the autosomal DNA is derived by the ST isoline (see Section 2; Larner et al., 2019). Preliminary experiments have shown that RasH1 and OuzM1 ST isolines show no suppression against SR (Verspoor et al., 2018), thus we used these isolines to produce the SR males that produce 100% daughters. Additionally, the introgression process was also done with an ST line from Tunisia, TabC38, that contains genes that are capable of suppressing SR. This Tunisian ST isoline was therefore used to produce the ^Supp^SR males that produce a normal 50:50 sex ratio despite the male carrying the SR X chromosome. After 12 generations of introgression, SR males with the susceptible genetic backgrounds produced a mean sex ratio across the three SR chromosomes of 99.4% ± 0.3 females. SR males that were introgressed onto the suppressing genetic background on the other hand produced a mean sex ratio of 50.5% ± 4.5 females, suggesting that these males were fully suppressed at the time the mate trials were conducted.

### 2.3 Mating performance

We compared male mating performance for nine types of males. Males carrying SR chromosomes from three geographic origins, those same SR chromosomes on a suppressive background, and three ST males (Appendix 1). Within the ST lines, TabC38 is the suppressing line in the absence of SR and RasH1 and OuzM1 are the susceptible lines in the absence of SR. The ST lines that were used were compared against each other to determine whether there were any effects of the suppressor in the absence of SR. We measured four components of male mating performance; mating success, copulation latency, copulation duration, and offspring production, in single‐male no‐choice mating trials. Mating latency, the time taken to achieve copulation, is a standard measure of precopulatory female choice in *Drosophila*, where preferred males are expected to achieve a mating more quickly (Avent et al., 2008; Prathibha et al., 2011; Somashekar & Krishna, 2011). Copulation duration, the amount of time a male spends copulating, is considered to reflect a male's investment in the mating (Bretman et al., 2011; Price, Lewis, et al., 2012; Price, Lizé, et al., 2012). Offspring production that occurred as a result of any successful mating was used as a further measure of male fitness. Mating success was also measured in additional trials where females were presented with a choice of two males. These two‐male mate trials may more closely resemble natural situations for wild flies, where males can actively compete for access to a female (Moore & Moore, 1999).

#### 2.3.1 Single‐male mating trials

To perform each mating trial, we separated 6‐day‐old males into individual vials (as male rivalry can affect mating latency; Lizé et al., 2014). After 24 h, each single male was added to a vial containing a random virgin female from our outbred Moroccan population (these females are healthy and robust and it did not make our results dependent on using females from a single inbred isoline) using an aspirator. Each male was used in only one mate trial. We recorded the time of male transfer into the female vial, the start of copulation, and the end of copulation. We observed males for a two‐hour window and if no mating was observed, we recorded the mating as unsuccessful. For each of the nine male types, we tested a total of 50 males in four experimental blocks over a two‐week period. Mate trials were blinded with one person adding flies to vials and the other recording the time of copulation. All trials started at 09:00 h GMT ± 5 min to coincide with dawn and occurred at 23°C (reported to be close to optimal for this species; Krimbas, 1993).

Following the mate trials, females were transferred into fresh vials of ASG double yeast medium and allowed to lay eggs for two periods of 7 days each. Larvae were allowed to develop into adults, and we counted the total emerging offspring as a further measure of male fitness. To standardize the eclosion window for offspring, each block was frozen 7 days after the first eclosion.

#### 2.3.2 Two‐male competitive mating trials

As geographical origin of SR had no strong effect on mating success in the single‐male mating trials, we pooled lines from different SR locations for the competitive mate trials but kept the suppressing and susceptible ST lines separate to continue to look for any effects of the suppressor in the absence of SR. Thus, for this experiment, there was a total of four male types (^Supp^SR – consisting of the three SR X chromosomes on the suppressing, TabC38, genetic background, ^Suscept^SR – consisting of the three SR X chromosomes on the susceptible, RasH1 or OuzM1, genetic backgrounds, ^Supp^ST – which are also the TabC38 males and ^Suscept^ST – consisting of RasH1 and OuzM1 males; see Appendix 2) and we tested all six combinations of males.

To differentiate males from each other, we used wing clipping. For each pair of males, we clipped the right wing tip of one male in the competitive pair when 2 days old (5 days before the trial) to minimize the effect of CO_2_ anesthesia (Barron, 2000; Verspoor, Heys, & Price, 2015). *D. subobscura* courtship is based on visual cues, rather than acoustic ones (Ewing & Bennet‐Clark, 1968; Ripfel & Becker, 1982) and so it is unlikely that the small amount of wing that we removed affected courtship behavior. To avoid mating bias, we clipped males' wings in a balanced design. When males were 7 days old, we added them to a vial containing a single virgin female (from our general population) and they were given a two‐hour window to mate. We recorded the wing‐clip phenotype of the successful male. We tested at least 50 replicates per competitive pair in four experimental blocks over a two‐week period. Mate trials were fully blinded and trials started at 10:00 h BST ± 10 min to coincide with dawn, at 23°C. Body size was accounted for by taking a wing measurement and comparing a subsample of mated and unmated males for 30 random competitive pairs per experimental block (Gilchrist et al., 2001).

### 2.4 Data analysis

For the single‐male mate trial, we used a binomial generalized linear model (GLM) to compare the mating success of males, Poisson GLMs to analyze mating latency and mating duration, and the number of offspring with a Gaussian GLM. All models considered male type (SR, ^Supp^SR, or ST), as well as the geographical origin of each line. For all GLMs, we produced a fully saturated model, which was reduced to the minimum adequate model through step‐wise reduction. Differences in responses to all GLMs under different treatments were assessed by analysis of variance (ANOVA) followed by a Tukey HSD comparison. We also ran all GLMs for only the ST lines to look specifically for effects of the suppressor genes in the absence of an SR chromosome.

For the two‐male mate trials, we used chi‐squared analyses on each competitive male pairing with an expected proportion of 0.5 in order to investigate if one male type could outcompete the other. The effect of wing clipping on female mate preference was also investigated using chi‐squared analyses. The body size effects on female mate preference were analyzed using a *t*‐test. All statistical analyses were done in R version 3.6.1 (R Development Core Team, 2019).

## 3 RESULTS

### 3.1 Single‐male mating performance

#### 3.1.1 Mating success

**FIGURE 2:**
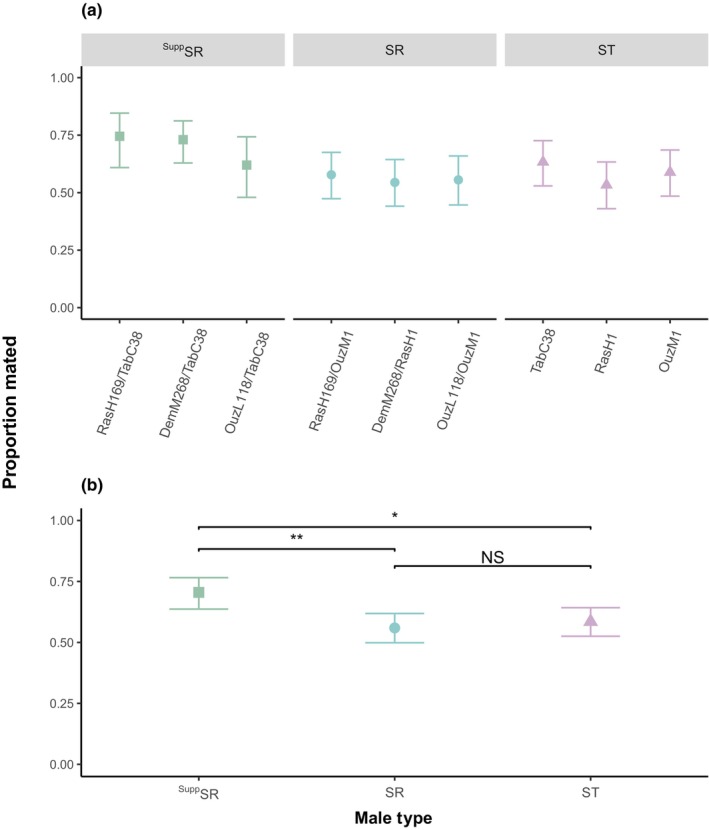
The proportion of males that gained a successful mating during the single‐male mate trial. Within the ST lines, TabC38 is the suppressing line in the absence of SR and RasH1 and OuzM1 are the susceptible lines in the absence of SR. (a) shows the mating success of males split by the geographic origin of each isoline used. Here, the term before the “/” is the origin of the SR X chromosome and after the “/” is the genetic background the chromosome is on. The ST lines have a standard X chromosome from the same location as their autosomes. (b) Mating success of males only considering the type of male. Here, ^Supp^SR (green) are males that carry a suppressed version of SR, SR (blue) are males that carry a normal driving version of SR and ST (purple) represents standard males that do not have an SR chromosome. The point represents the mean and the error bars are the 95% confidence intervals. The figure reports Tukey HSD comparisons where NS is non‐significant, * is *p* < .05, and ** is *p* < .01.

We compared the mating success of males that carried SR, ^Supp^SR, or ST males. We analyzed the data using the following statistical model to determine what factors contributed to mating success: mating success as a function of male type (whether a male was SR, ^Supp^SR, or ST) + geographical origin of the X chromosome. The geographic origin of the SR chromosome had no effect on mating success (Figure 2a: GLM: *F*_6,712_ = 0.708, *p* = .644), and so was dropped from the mating success model. We also tested whether there were any differences in mating success within the ST lines to see if the genetic backgrounds could alter mating success in the absence of SR. There was no variation in mating success within the ST lines (Figure 2a, GLM: *F*_2,267_ = 0.927, *p* = .397), indicating the suppressor genes in the absence of drive (TabC38) did not have an effect on mating success rate. Mating success was significantly affected by male type (Figure 2b: GLM: *F*_2,718_ = 5.415, *p* < .01), where suppressing the drive improved mating success. Males carrying a suppressed version of SR had a 71% success rate, significantly higher than the 56% success rate of unsuppressed SR males (Tukey HSD, *p* < .01) and 59% success rate of ST males (Tukey HSD, *p* = .025). There was no difference in the mating success of SR and ST males (Tukey HSD, *p* = .814).

#### 3.1.2 Copulation latency

**FIGURE 3:**
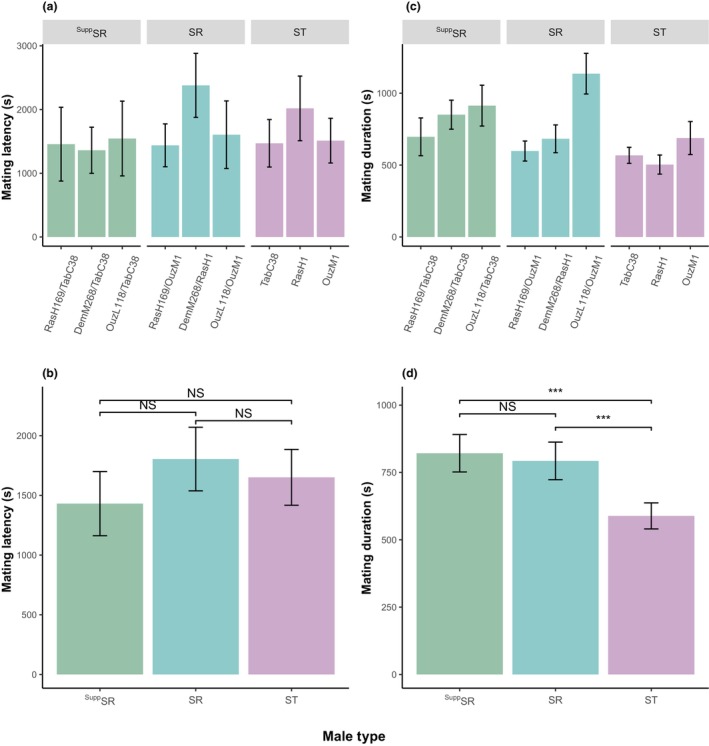
Bar plots showing the differences in (a) and (b) copulation latency (in seconds) and (c) and (d) copulation duration in seconds for suppressed SR (^Supp^SR), SR, and standard (ST) males. Within the ST lines, TabC38 is the suppressing line in the absence of SR and RasH1 and OuzM1 are the susceptible lines in the absence of SR. (a) and (c) show the individual isolines used in the mate trials. Here, the term before the “/” is the origin of the SR X chromosome and after the “/” is the genetic background the chromosome is on. The ST lines have a standard X chromosome from the same location as their autosomes. (b) and (d) only considers the type of male and whether they carried a version of SR. Here, ^Supp^SR are males that carry a suppressed version of SR, SR are males that carry a normal driving version of SR and ST represents standard males that do not have an SR chromosome. The error bars show the 95% confidence intervals. The figure reports Tukey HSD comparisons where NS is non‐significant and *** is *p* < .001.

We measured copulation latency as the time it took for a male to gain a mating. We analyzed copulation latency using a model including male type (whether a male was SR, ^Supp^SR, or ST) and geographical origin of the X chromosome as factors. The geographic origin of the SR chromosome had a significant effect on copulation latency (Figure 3a: GLM: *F*_8,430_ = 2.342, *p* = .018), with the susceptible SR type originating from Demnat (DemM268/RasH1) taking significantly longer to mate than DemM268/TabC38 (Tukey HSD: *p* = .016). There were no differences in copulation latency among the three ST lines (Figure 3a: GLM: *F*_2,155_ = 2.143, *p* = .121). We also found no significant difference in the time taken for males to gain a copulation between the three male types (Figure 3b: GLM: *F*_2,430_ = 2.052, *p* = .130).

#### 3.1.3 Copulation duration

We measured copulation duration by timing how long individual matings lasted, and analyzed this data using the following model: copulation duration as a function of male type (whether a male was SR, ^Supp^SR, or ST) + geographical origin of the X chromosome. Both the origin of the SR chromosome and the male type significantly affected mating duration (Figure 3c: GLM: *F*_6,427_ = 13.03, *p* < .001 and Figure 3d: GLM: *F*_2,427_ = 19.56, *p* < .001, respectively). Overall ^Supp^SR and SR mated for longer than ST males (^Supp^SR and SR mean mating time 821 seconds ±35 and 793 ± 35, respectively, vs. ST males mean of 589 seconds ±24; Tukey HSD: *p* < .001, Tukey HSD: *p* < .001, respectively). There was no difference in the mating duration between ^Supp^SR and SR males (Tukey HSD: *p* = .780). Within the three male types, we also found significant variation among the isolines, these differences appear to be idiosyncratic and follow the main drive effects. There was some significant variation in the mating duration of the ST isolines (Figure 3c: GLM: *F*_2,155_ = 4.990, *p* < .01), where the two susceptible ST lines mated for different durations (OuzM1 mated for longer than RasH1, Tukey HSD: *p* < .01).

#### 3.1.4 Offspring production

**FIGURE 4:**
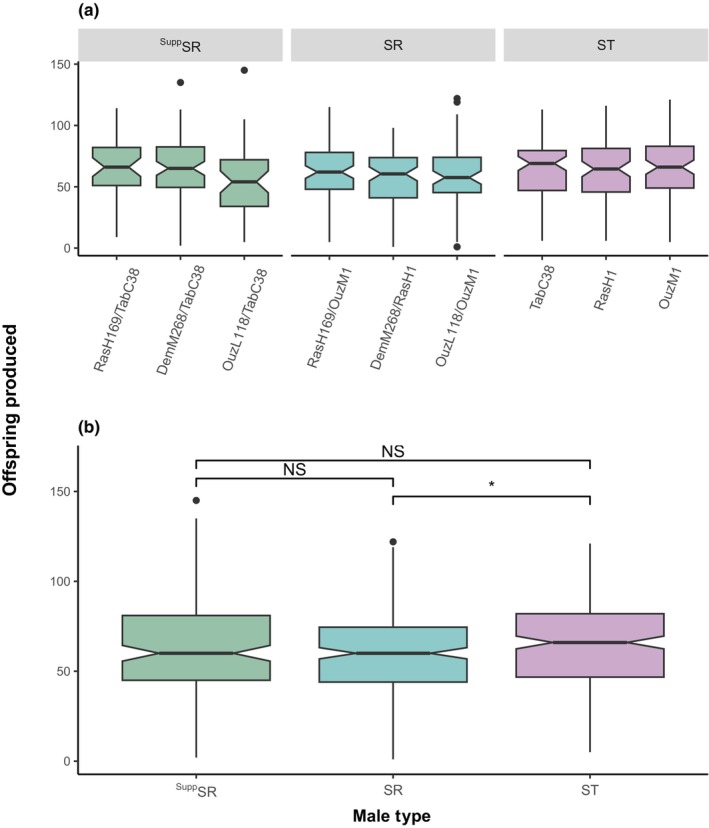
Number of offspring produced after successful matings in single‐male mate trials. Within the ST lines, TabC38 is the suppressing line in the absence of SR and RasH1 and OuzM1 are the susceptible lines in the absence of SR. (a) considers the offspring production of males as well as the location of each isoline used. Here, the term before the “/” is the origin of the SR X chromosome and after the “/” is the genetic background the chromosome is on. (b) considers the offspring production of males only considering the type of male and whether they carried a version of SR. Here, ^Supp^SR are males that carry a suppressed version of SR, SR are males that carry a normal driving version of SR, and ST represents standard males that do not have an SR chromosome. The boxplots display the upper and lower quartiles, the median, and the range and the notches represent the 95% confidence intervals. The figure reports Tukey HSD comparisons where NS is non‐significant and * is *p* < .05.

We counted the number of offspring produced as a result of each mating as a further measure of male fitness. We analyzed offspring production, using a GLM including male type (whether a male was SR, ^Supp^SR, or ST) and geographical origin of the X chromosome as factors. We found no differences in number of offspring produced by the three ST male isolines (Figure 4a: GLM: *F*_2,621_ = 0.797, *p* = .452). The geographic origin of SR did not have an effect on offspring production (Figure 4a: GLM: *F*_6,639_ = 1.514, *p* = .171); however, male type did have a significant effect on offspring production (Figure 4b: GLM: *F*_2,639_ = 3.498, *p* = .031). Females that mated with ST males produced significantly more offspring than SR males (ST males produced a mean of 66 offspring compared to 58 from SR males; Tukey HSD: *p* = .024), whereas when females were mated with ^Supp^SR males, they produced a mean of 62 offspring, which did not differ from SR males (Tukey HSD: *p* = .309) or ST males (Tukey HSD: *p* = .624).

### 3.2 Two‐male competitive mating trial

We independently retested the mating success patterns we found in single‐male mate trials by exposing different combinations of male types to a single female in two‐male competitive mate trials. For all competitive trials, the two males competing against each other were distinguished from one another by clipping one of the male's wing, which did not affect female‐mating preference (Appendix 3a: *χ* ^2^ = 0.997, df = 1, *p* = .318). Additionally, we also tested whether male body size influenced which male was successful out of the competitive pair, however, we found no significant difference between the body size of males that gained the mating and the unmated males (Appendix 3b: *t* = 0.076, df = 233.79, *p* = .940).

**FIGURE 5:**
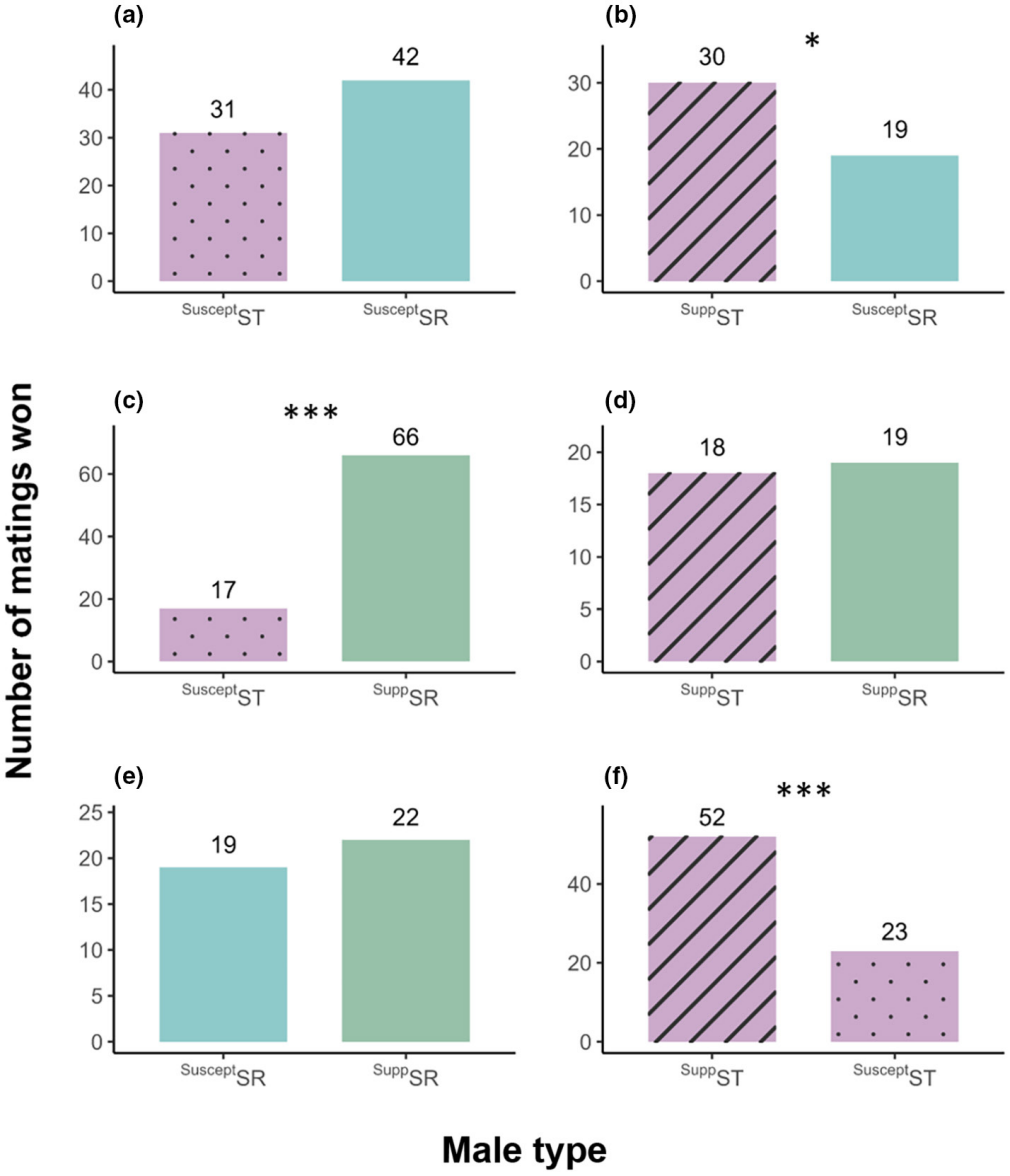
Number of matings won by each male type in a two‐male competitive mating trial. Here, ^Supp^ST males are the TabC38 males from the single‐male mating trials and the ^Suscept^ST males are a combination of RasH1 and OuzM1 males from the single‐male mating trials. (a–f) are the different combinations of male types competing against each other in the mate trials for access to one female and shows the number of matings won by each male type when competing against the other. The superscript denotes the genetic background the X chromosome is on; either susceptible (Suscept) or suppressing (Supp) and the main term denotes the type of X chromosome; either ST or SR. Blue represents SR males, green represents ^Supp^SR males and ST males with suppressing genes were purple with stripes, and ST males with susceptible genes are purple with dots. The figure reports significant chi‐squared results where * is *p* < .05, and *** is *p <* .001.

In general, males that carried the suppressor background outcompeted males without the suppressor (Figure 5). This was true within ST‐ST comparisons, where ST‐suppressing males successfully outcompeted ST‐susceptible (Figure 5f: *χ* ^2^ = 20.907, df = 1, *p* < .001). This was also true when comparing SR and ST males: ST‐suppressor was more successful than SR (Figure 5b: *χ* ^2^ = 4.082, df = 1, *p* = .043), and ^Supp^SR were more successful than ST‐susceptible males (Figure 5c: *χ* ^2^ = 55.518, df = 1, *p* < .001). Surprisingly though, the comparison between the ^Supp^SR and SR was not different (Figure 5e: *χ* ^2^ = 0.195, df = 1, *p* = .659).

With suppressor or susceptible background held constant within the competitive pair, there were no differences between SR and ST flies. When both males the carried suppressor background (ST‐suppressing males competing against ^Supp^SR males), the two male types gained an equal number of matings (Figure 5d: *χ* ^2^ = 0.000, df = 1, *p* = 1.000). Also, when both males lacked the suppressor genes, ST and SR males also did not win significantly different numbers of matings (Figure 5a: *χ* ^2^ = 2.740, df = 1, *p* = .098).

## 4 DISCUSSION

Despite an unfair transmission advantage, the SR chromosome of *D. subobscura* has failed to spread to a high frequency in the wild, and instead it occurs at the same substantial but low frequency (~20%) in the wild as it did, when it was discovered, in the 1960s (Hauschteck‐Jungen, 1990; Jungen, 1967; Verspoor et al., 2018). As wild‐caught males express drive (Hauschteck‐Jungen, 1990; Jungen, 1967; Verspoor et al., 2018), suppression has also failed to spread through natural populations of *D. subobscura*. One explanation for why these phenotypes have failed to spread is that they may carry costs which counterbalance their advantages. The costs of drive chromosomes have been described in other systems (Angelard et al., 2008; Hamilton, 1967; Keais et al., 2020), but any costs of suppression have yet to be studied.

Here, we investigate potential costs to SR and its suppressor associated with mating and reproduction. For *D. subobscura*, mate discrimination may be especially important, as females of this species are monandrous (Fisher et al., 2013; Verspoor et al., 2016). Mate discrimination against drive‐carrying males is found in stalk‐eyed flies, where male attractiveness and genetic quality are linked to eye span size (David et al., 1998; Panhuis & Wilkinson, 1999). The stalk‐eyed fly XCMD SR chromosome is linked to males having smaller eye spans and reduced attractiveness (Cotton et al., 2014; Wilkinson et al., 1988). Such a system allows females to avoid mating with SR males, thus also avoiding the costs that SR imposes (Finnegan et al., 2019).

In *D. subobscura*, we found that females did not discriminate against SR‐bearing males, either in single‐male or competitive mate choice trials, whether using mating success or mating latency as the measure of male attractiveness. This lack of discrimination against unsuppressed SR males is consistent with other studies of XCMD systems in *Drosophila* (Price, Lewis, et al., 2012; Price, Lizé, et al., 2012; Price & Wedell, 2008), including previous work in *D. subobscura* (Verspoor et al., 2016). A deleterious XCMD system that can be detected by females is likely to be rapidly selected against and so there may strong selection for SR to be indistinguishable, as previously suggested (Price, Lewis, et al., 2012).

We also found that females did not discriminate against males carrying a suppressor – in fact, surprisingly, we found some evidence that females prefer to mate with suppressor‐carrying males. As this effect also occurred independently of SR, it suggests that this is directly due to the suppressor or linked genes, rather than its interaction with SR. How this suppressor affects mating success, and whether this effect is male‐ or female‐driven is unclear. If female‐driven, it may be that the suppressor background, which was derived from one strain, contains alleles that favorably affect attractiveness. Alternatively, if male‐driven, the suppressed males may have increased their reproductive effort due to reduced reproductive value — this “terminal investment” in reproduction (Williams, 1966) has been shown in other contexts (Brannelly et al., 2016; Creighton et al., 2009; Velando et al., 2006). It is possible that this result is driven by the poor performance of susceptible control males in competitive mate choice trials. However, this explanation seems unlikely: in single‐mate trials, susceptible males performed similarly to other types of males, and the three isolines performed similarly to each other, suggesting they are representative. In any case, this result suggests that the suppressor does not have a strong negative effect on mating success, and is not a major factor preventing the spread of suppression.

In contrast to mating, we found a large cost for unsuppressed SR males in offspring production, which was reduced by 12% in comparison to ST males. On its own, this reduction in offspring production is unlikely to be enough to overcome the transmission advantage of SR. Our measurement of offspring production, however, occurs in males mating with a single female. With multiple matings, SR males, who suffer the loss of half their sperm, may suffer a larger reduction in offspring number. In fact, there was some indication that SR males are sperm‐limited, as they had increased mating durations in comparison to ST (see also Verspoor et al., 2016). Increased mating duration may indicate and compensate for inefficient and a lower quantity of sperm transferred.

The reduction in offspring number thus could be due to lower rates of sperm transfer and lack of fertilization. Consistent with this idea, suppressed SR males, who do not suffer the loss of Y‐bearing sperm, had intermediate numbers of offspring. However, why suppressed SR males did not produce an equivalent number of offspring to ST is puzzling, as the suppressor should rescue all of a males' Y sperm (Price et al., 2020). Although, if SR causes a delay to the production of sperm, a suppressor may only be partly effective at preventing this. That said, the reduction in offspring of SR males could also be wholly or in part due to a higher incidence of embryo/larval death, which would likely be due to deleterious alleles linked to SR rather than SR itself.

Overall, we found that SR‐carrying males were equally as attractive as wild‐type males, suggesting discrimination against carriers does not counter the spread of SR. We also found the first evidence that carriers of a suppressor of SR have higher mating success, both when paired with SR and in its absence, suggesting mate discrimination does not explain why the suppressor is rare. We did find some evidence that SR males may be sperm limited, consistent with these males losing half of their sperm: SR‐carrying males have both an increased mating duration and lower offspring production. The effects that we measured appear not to be strong enough to counter‐drive on their own, although they may act alongside other stabilizing factors.

## AUTHOR CONTRIBUTIONS

**Sophie Lyth:** Conceptualization (lead); data curation (lead); formal analysis (lead); investigation (lead); methodology (equal); validation (equal); visualization (lead); writing – original draft (lead); writing – review and editing (lead). **Andrea J. Betancourt:** Conceptualization (supporting); formal analysis (supporting); supervision (equal); visualization (supporting); writing – original draft (supporting); writing – review and editing (supporting). **Tom A. R. Price:** Conceptualization (supporting); formal analysis (supporting); funding acquisition (supporting); methodology (equal); supervision (equal); writing – original draft (supporting); writing – review and editing (supporting). **Rudi L. Verspoor:** Conceptualization (equal); formal analysis (supporting); investigation (supporting); methodology (equal); supervision (equal); validation (equal); visualization (supporting); writing – original draft (supporting); writing – review and editing (supporting).

## Data Availability

Data used for this manuscript can be found through the following https://doi.org/10.5061/dryad.mcvdnck61.

## APPENDIX 1

### 1.1

The nine male types used in the single‐male mating trials. For SR and ^Supp^SR, the first part of the line code is the origin of the SR chromosome and the second part is the genetic background it was introgressed onto. ST male types consider the mating ability of these lines in the absence of drive.

**Table:**

SR males	^Supp^SR males	ST males
RasH169/OuzM1	RasH169/TabC38	OuzM1 (susceptible)
DemM268/RasH1	DemM268/TabC38	RasH1 (susceptible)
OuzL118/OuzM1	OuzL118/TabC38	TabC38 (suppressing)

## APPENDIX 2

### 2.1

The four male types used in the two‐male mating trials. For SR and ^Supp^SR, the first part of the line code is the origin of the SR chromosome and the second part is the genetic background it was introgressed onto. ST male types consider the mating ability of these lines in the absence of drive that can either suppress SR or are susceptible to it.

**Table:**

SR males	^Supp^SR males	ST suppressing	ST susceptible
Consisting of: RasH169/OuzM1, DemM268/RasH1, OuzL118/OuzM1	Consisting of: RasH169/TabC38, DemM268/TabC38, OuzL118/TabC38	Consisting of: TabC38	Consisting of: RasH1, OuzM1

## APPENDIX 3

### 3.1

(a) The number of matings won by clipped and unclipped males in the two‐male mating trials. (b) The wing length of males that won or lost the mating in the two‐male competitive mating trials. The boxplots display the upper and lower quartiles, the median, and the range.
